# A multilevel analysis of financial institutions’ systemic exposure from local and system-wide information

**DOI:** 10.1038/s41598-020-74259-7

**Published:** 2020-10-19

**Authors:** Yérali Gandica, Sophie Béreau, Jean-Yves Gnabo

**Affiliations:** 1 Center for Research in Finance and Management (CeReFiM), University of Namur, Namur, Belgium; 2 Namur Institute for Complex Systems (naXys), University of Namur, Namur, Belgium; 3 Center for Operations Research and Econometrics (CORE), Université catholique de Louvain, Ottignies-Louvain-la-Neuve, Belgium; 4 Institute of Information and Communication Technologies, Electronics and Applied Mathematics (ICTEAM), Université catholique de Louvain, Ottignies-Louvain-la-Neuve, Belgium; 5 CY Cergy Paris Université, CNRS, Laboratoire de physique théorique et modelisation, 95000 Cergy, Île-de-France France

**Keywords:** Mathematics and computing, Physics

## Abstract

In the aftermath of the financial crisis of 2007–2009, the growing body of literature on financial networks has widely documented the predictive power of topological characteristics (e.g., degree centrality measures) to explain the systemic impact or systemic exposure of financial institutions. This study shows that considering alternative topological measures based on local sub-network environment improves our ability to identify systemic institutions. To provide empirical evidence, we apply a two-step procedure. First, we recover network communities (i.e., close-peer environment) on a spillover network of financial institutions. Second, we regress alternative measures of vulnerability (i.e. firm’s losses)on three levels of topological measures: the global level (i.e., firm topological characteristics computed over the whole system), local level (i.e., firm topological characteristics computed over the community to which it belongs), and aggregated level by averaging individual characteristics over the community. The sample includes 46 financial institutions (banks, broker-dealers, and insurance and real-estate companies) listed in the Standard & Poor’s 500 index. Our results confirm the informational content of topological metrics based on a close-peer environment. Such information is different from that embedded in traditional system-wide topological metrics and can help predict distress of financial institutions in times of crisis.

## Introduction

In the aftermath of the 2007–2009 financial crisis, which was characterized by isolated shocks to specific financial institutions spreading to the entire system, with the Lehman Brothers default in Autumn 2008 standing out as the most remarkable illustration, it has become crucial to better understand how contagion episodes operate within the financial system. Since then, increasing attention has been devoted to exploring and measuring financial risk at the system-wide level, the so-called “systemic risk.” The aim is to guide policy makers in designing adequate macro-prudential regulation instruments and detect systemically important financial institutions (SIFIs). With the paradigm shift from a micro- to a macro-prudential perspective, the financial industry is being viewed as an emergent system and as such, its properties can be revealed by means of tools from network science. In this framework, financial institutions are assimilated to the nodes of the network and the connections between them are represented by the edges. The latter can be financially motivated as is the case for cross-lending between banks or simply the outcome of unknown mechanisms resulting in statistical dependence across institutions’ stock returns. Based on this stylized representation, a common method consists of applying centrality measures to flag risky institutions. The convention is to consider institutions with numerous outgoing links as risky for the system, and those with numerous incoming links as fragile institutions. The analysis has been extended to other centrality measures such as Bonacich, Katz, or Betweenness centrality (e.g., see^1^).

While substantial efforts in empirical financial networks have been devoted to retrieving unobserved connections, less effort has been made in terms of signal extraction once the network is formed. We argue that centrality measures commonly used in the literature and applied at the network level ignore auxiliary information embedded in the sub-part of the network. Such information appears once the whole system is segmented into smaller-scaled and more local environments consisting of more densely connected nodes. These environments are often called *communities* and emanate from privileged relationships among certain financial institutions or cross-holdings. For example, they have the ability to alleviate the transmission of shocks through feedback effects, which is of crucial importance for risk assessment. However, to the best of our knowledge, no existing research relies on centrality measures by combining information at the intra-community level along with the usual system-wide level. The current study empirically explores this issue. To this end, we rely on the spillover network of^2^ based on a time-varying parameter vector autoregressive (TVP-VAR) model as well as Granger causality statistical tests on stock market returns to recover the unobserved spillover network of financial institutions. From there, we use the Louvain algorithm to identify the different communities constituting the network. This step is crucial to construct a new set of centrality metrics based exclusively on close-peer information. Next, we compute topological characteristics at three different levels: (i) the global level (i.e., firm topological characteristics computed over the whole system), (ii) local level (i.e., firm topological characteristics computed over the community), and (iii) aggregated level by averaging individual characteristics over the community. We refer to them as global topological metrics (GT), local topological metrics (LT) and aggregated topological metrics (AT), respectively. To assess the informational content of each measure, we apply the elastic net regression technique and test whether it can explain bank losses during the financial crisis. Our sample includes 46 financial institutions including banks, broker-dealers, and insurance and real-estate companies listed on the Standard & Poor’s (S & P) 500 index. Our sample is similar in size or larger than most related studies on market-based financial spillovers.

## Literature review

Our study relates to the fast growing literature on contagion in financial networks (see^3^ for a survey). A central question in this body of research is whether the network structure enhances the vulnerability of the financial systems as a whole and of its components. The degree distribution is a common measure to characterize the network structure; for example, Boss et al.^4^ document this question using real data on interbank liabilities in Austria. A similar exercise is proposed in^5^ and^6^ for the Brazilian interbank network. Caldarelli et al.^7^ argue that, as in other types of networks such as social media, degree distributions in financial networks are found to be well described by a power law. An interesting feature emerging from this research is the identification of core–periphery structure in financial market especially in interbank networks. Thereby, the system is comprised of a small number of highly interconnected banks at the core along with poorly connected institutions on the periphery (see^8^). Part of the literature shifts the perspective from the system to individual nodes and extends the analysis to centrality measures that are designed to identify the importance of a node in a network (see^9^). One of the primary targets of this research is then to assess whether the centrality of nodes explains their financial exposure or systemic importance. To document this issue, several centrality measures used in network science have been applied to financial systems such as degree, eigenvector, or Katz centrality to cite the most frequent. For example, Craig et al.^10^ apply a modified version of the eigenvector centrality on German credit registers to explain individual bank risk. They find a negative relationship between centrality measures and the probability of default. In^11^, Martinez-Jaramillo et al., compute several centrality measures into a composite measure of Mexican data to characterize the banking system. Puhr^12^ find a positive relationship between the Katz centrality and systemic risk. In these examples, centrality measures are applied on single layer networks implicitly assuming a unique source of connection between financial institutions, such as contractual obligation. However, the reality is more complex. Potential transmission channels are diverse^13,14^ such as cross-lending relationships, derivatives, similarities, or common holdings in the portfolio structures, among others^15^. Each channel in turn may create a specific network of dependence among the financial institutions at stake. To feature the different layers, one strand of the literature has developed a holistic approach that considers multiple channels of transmission, while keeping the network representation simple. To this end, the links are recovered from the analysis of the dependence structure in stock market returns. The approach builds on the premise that stock prices reflect all the relevant information regarding an institution. As such, the dependence between stock returns enables us to assess whether two institutions are related, regardless of the specific channels through which the transmission operates. Thereby, it provides a synthetic measure of interconnectedness between institutions. In this vein, Billio et al.^16^ has made a pioneering contribution. The author uses the Granger causality test over monthly returns of hedge funds, banks, broker/dealers, and insurance companies to build their network. It is then possible to predict the systemic risk level of financial institutions out-of-sample based on centrality measures that are computed on the retrieved network structure. Diebold and Yilmaz, Giudici and Parisi, Betz et al.^17–19^ performed similar studies. Gandica et al.^20^ document the presence of sub-structures within the network of densely connected nodes. Sub-networks across time in the US market were identified using a community detection algorithm. An interesting finding of this research is that these sub-networks stemming from stock returns dependence do not fully coincide with trivial ex-ante categories such as financial industries (e.g., broker-dealers vs. banks). It should be noted that, while being different, this approach shares similarities with a related strand of the literature where stock market returns can also be used to compute systemic risk measures based on commonalities in the market as a whole (e.g., see^21^ or the PCA analysis in^16^).

Our contribution is closely related to the analysis developed in^16^. The main differences are threefold. First, we rely on a modified procedure, as developed in^2^, to retrieve the financial network. As discussed in^2^, this approach is better suited when the underlying network is time varying. Second, we segment our network into sub-networks by applying a community detection algorithm. Third, we compute topological measures at three levels: (i) the firm level over the whole system as in^16^, (ii) the firm level over the community, and (iii) the community level by averaging individual characteristics within identified communities. Another related contribution is that of^1^, who examine the exploratory power of a large set of centrality measures of interbank spreads.

## Dataset

Our empirical analysis of firms’ systemic risk crucially depends on two main elements: (i) an accurate representation of the underlying pre-crisis financial network. The ability to properly assess relevant network metrics that will serve as explanatory variables in our regression settings also depends on this. (ii) A sound measure of systemic risk in times of crisis (our endogenous variable). To address both issues, we rely on a purely market-based approach and retrieve the monthly cum-dividend stock prices from Thomson Reuters Eikon for each company in a sample covering the period from January 1990 to December 2014 for the former and over the crisis period (July 2007–December 2008) for the latter. We derive our financial spillover network data using the methodology developed by^2^. The data feature the time-varying bilateral relationships of 155 financial institutions with Standard Industrial Classification (SIC) codes from 6000 to 6799 from the S&P 500 over the pre-crisis period (more details about the creation of the network based on raw return data are provided on the supplementary information). Three subsequent filters on this network were needed to recover the final network on which pre-crisis topological measures are computed. First, institutions appearing in the sample only after the start of the financial crisis in January 2008 were removed. Second, to filter out the noise in our data, the analysis is restricted to stocks with at least 36 consecutive monthly observations. Eventually, the financial institutions that disappear before or during the financial crisis were dropped. Our final sample consists of 46 financial institutions including banks, broker-dealers, and insurance and real-estate companies listed on the S & P 500 index. Note that such a network is similar in size or larger than most related studies on market-based financial spillovers such as^22,23^, or^24^. Based on the resulting pre-crisis network representation, network metrics are computed at various levels (firm-global, firm-local, community), as detailed in Step 2 below. Finally, systemic risk metrics are detailed in Step 3, based on transformations of either raw prices or log-returns in times of crisis.

## Methodology

First, we recover the financial network for the set of institutions in our sample. Second, based on a community detection algorithm, we break down the whole network into sub-networks, and compute pre-crisis topological measures at both the system-wide and community levels. Third, we derive a measure of vulnerability in times of crisis. Fourth, we regress our measure of vulnerability on previous standard topological measures along with community-based ones to assess their marginal explanatory power. Each step is detailed below.

### Step 1: Financial temporal networks

**Figure 1:**
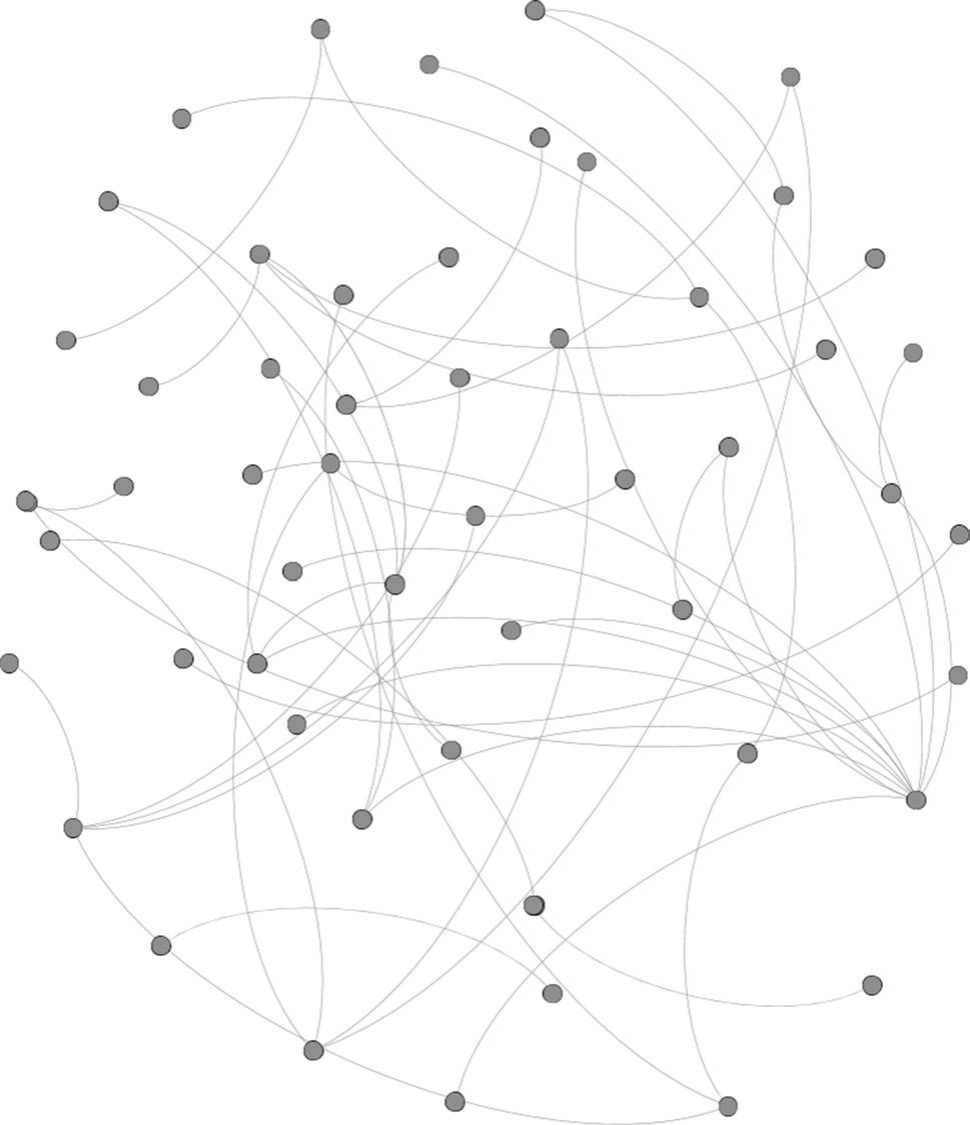
Pre-crisis financial network estimated by TVP-VARs. The figure features an illustrative snapshot of the network estimated by TVP-VARs for a pre-crisis period (September 2006). Nodes represent the financial institutions of our sample and edges their relationships as revealed by the Granger-causality testing over the time series of their respective log-returns (see the supplementary information or^2^ for more details).

Our financial networks were built following^2^ (see the supplementary information or^2^ for more details on the computation of the network based on raw return data). The linkages between financial institutions are retrieved from stock market returns by means of a Bayesian time-varying VAR framework and Granger causality testing procedures. According to this approach, an incoming link is created from institution *j* to institution *i* if the time series associated with the return on firm *j* Granger causes the time series of the return on firm *i*. Figure 1 provides an illustrative snapshot of the network before the 2007–2009 crisis. The details of the methodology can be found in^2^, and the main features are summarized in the supplementary information.

### Step 2: Independent variables

In a second step, we break down the whole network into sub-networks, grouping the most connected nodes together. To this end, we apply the Louvain method^25^, which is designed to detect communities within complex networks. This well-known method relies on a greedy optimization algorithm that attempts to optimize the modularity of a partition of the network, which is the strength of division of a network into modules or communities with high modularity capturing dense connections between the nodes within modules but sparse connections between nodes in different modules. The optimization is performed in two steps. First, the method looks for “small” communities by optimizing modularity locally. Second, it aggregates nodes belonging to the same community and builds a new network whose nodes are the communities. These steps are repeated iteratively until a maximum of modularity is attained and a hierarchy of communities emerges (for further details, see^25^).

**Figure 2:**
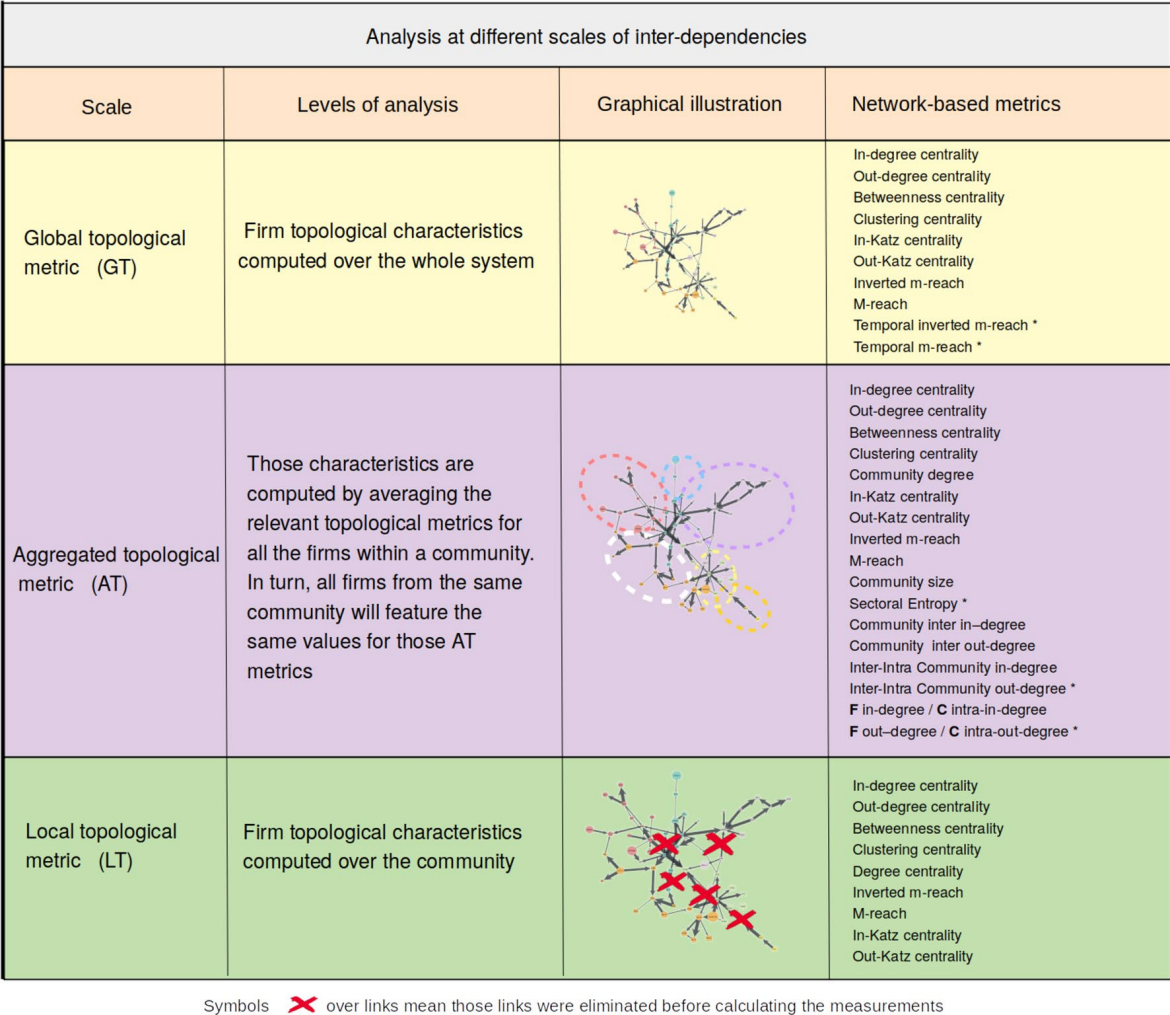
Three levels of analysis: System wide firms-based metrics, Community firm-based metrics and Community-based metrics. The figure displays the labels of the topological metrics along with their corresponding level. We consider three levels. System wide firms-based metrics are computed for each firm based on information stemming from the whole network. Community firm-based metrics are computed for each firm based on information stemming exclusively from the community (i.e. sub-networks). Community-based metrics are computed at the community level. Each category is associated to a stylized network to illustrate which area is used to compute the metrics. Metrics surmounted by an $^*$ correspond to the variables labelled “new” in the regression sections from each relevant category.

Equipped with a clearly identified community structure, we compute several topological measures by considering the whole network or only the community-based local environment. For the sake of clarity, the entire set of variables is divided into three groups. First, *global topological* metrics are computed at the firm level over the whole system, as is usually the case in the literature. Second, we compute the *local topological* metrics at the firm level over close peers. Unlike the previous metrics, for each node, we exclusively consider the other nodes within the same community when computing our metrics, thereby limiting topological metrics to intra-community links. Third, to further explore the influence of the local environment, we average individual characteristics over each community to compute *aggregated topological* metrics. This means that all the nodes included in the same community display similar values for this set of variables. Turning to the topological metrics we are considering, we account for a wide set of standard measures: *in-degree centrality*, *out-degree centrality*, *betweenness centrality*, *clustering centrality*, *m-reach centrality*, *inverse m-reach centrality*, *in-Katz centrality*, and *out-Katz centrality* (detailed explanations of the variables can be found in^20^). To take advantage of historical changes in the network as well as auxiliary information on firms’ industries, we complete our analysis with a set of less conventional metrics. Hence, sectoral entropy assesses how diverse a community is in terms of sectors. Inter in(out)-degree depicts the number of in(out)-degrees between communities. The inter-intra degree is computed as the ratio of the inter-community degree over the number of intra-community degrees. The measure is applied separately to in- and out-degrees. In addition, we compute the ratio between a firm’s in(out)-degree and its community in(out)-degree as a measure of the node’s commitment^26^. Finally, two temporal metrics are added. To this end, we simply compute a modified m-reach measure while considering that the contagious process involves a time lag. For example, if a firm displays an out-degree of 3 at time *t*, the value for its 1-reach centrality is 3. For the 2-reach centrality measure, we consider the connections of the 3 neighboring nodes at $t+1$ instead of time *t* in the usual case. If this value is equal to 4, the 2-reach centrality measure is 7. The measure takes a 1-period delay at each order of the propagation process into account. We consider a separate measure for both incoming and outgoing links. Figure 2 provides an overview of the variables constructed for the empirical analysis.

### Step 3. Dependent variables

**Figure 3:**
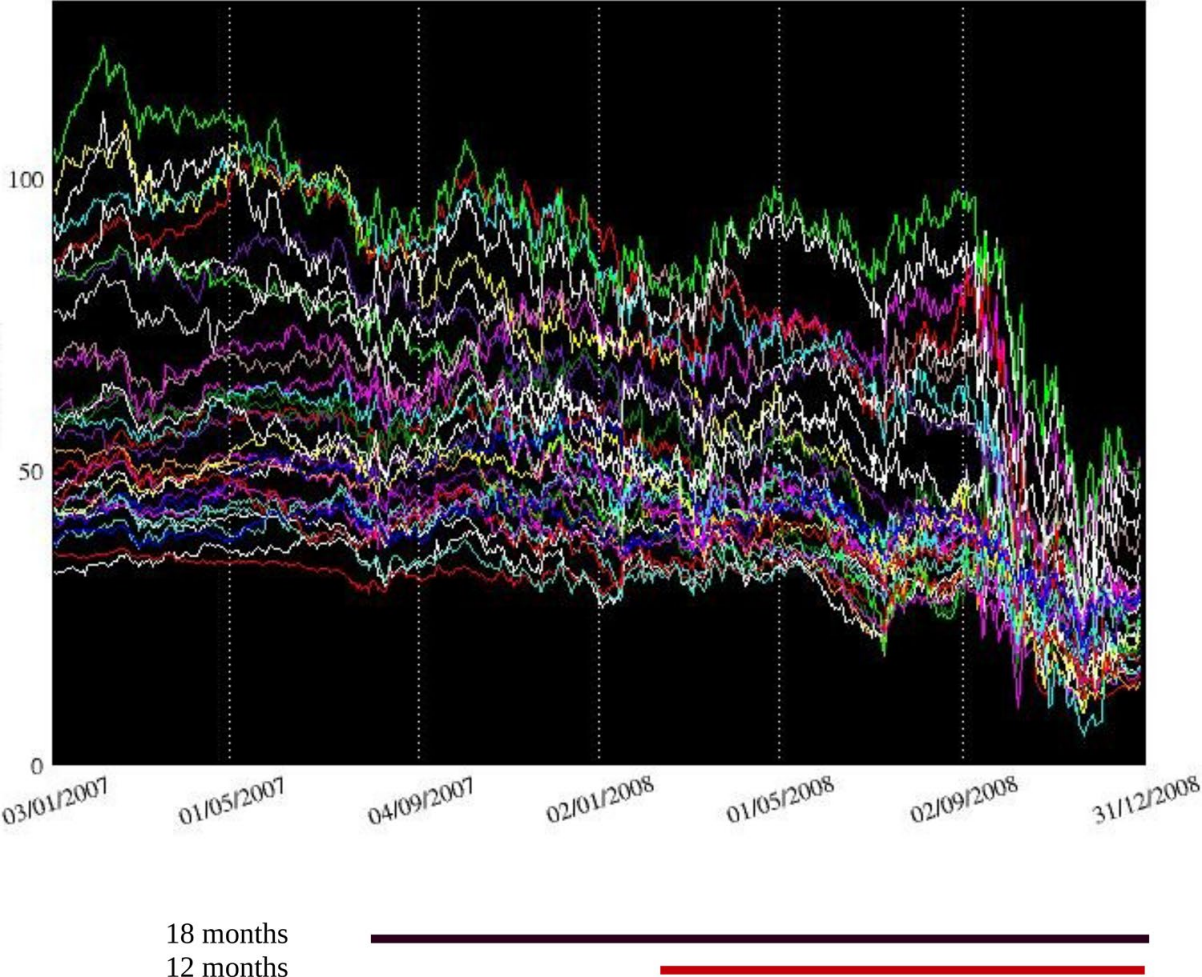
Stock market prices of financial institutions between 1/3/2007 and 12/31/2008. The figure presents the evolution of daily stock market prices of the 47 US banks included in our sample. The straight lines at the bottom of the figure correspond to the alternative time periods we use—July 2007 to December 2008 and January 2008 to December 2008—to define the crisis.

Following^24^, we use two indicators of firm vulnerability based on the loss in firm’s stock value to test the predictive power of our topological measures: (i) the cumulative stock returns, and alternatively (ii) the peak-to-trough returns. The former measure is based on daily log-returns and the latter on prices. The cumulative returns are computed for each firm as the sum of its stock returns over the crisis period. The maximum drawdown is computed as the maximum loss of the stock price over the crisis period from a peak to a trough, with the trough defined as the minimum value of the price between the date of the peak to the end of the crisis. As in the literature, we build on the premise that firms’ vulnerability is better revealed in the crisis period. The crisis can therefore be viewed as a natural experiment to identify vulnerable institutions and test whether such fragility could have been detected prior to the event by looking at alternative characteristics such as topological metrics. For robustness purposes, we use two definitions for the crisis period: (i) July 2007 to December 2008 and (ii) January 2008 to December 2008. Figure 3 displays the daily stock market prices for all the firms of our sample between 2007 and 2008. The alternative time periods to define the crisis are marked with a straight line at the bottom of the figure. A brief overview reveals that financial firms experienced their largest losses during the second period (January 2008 to December 2008).

### Step 4: Penalized regression analysis

Given the large number of regressors, we apply the elastic net (EN) model^27^ to perform shrinkage and identify the most relevant variables^28^. Along with the LASSO approach, this has been widely used in the literature to control the degree of sparsity in the model and select only the most significant coefficients^29^. The objective function in EN regression is expressed as follows:

1 $$\begin{aligned} min_{\beta _0,\beta } \left\{ \frac{1}{N} \sum _{i=1}^{N} ( y_{i} - \beta _0 - \beta ^T x_{i})^2 + \lambda [(1- \alpha ) ||\beta ||_2^2 / 2 + \alpha ||\beta ||_1] \right\} , \end{aligned}$$

The EN penalty is controlled by the mixing parameter $\alpha$, averaging two mainstream shrinkage penalty functions, namely the one referring to the LASSO (L1 penalty)^30^ and that associated with the ridge regression (L2 penalty)^31^. Note that if $\alpha =1$, the EN approach falls into the LASSO selection model whereas if $\alpha =0$, we recover a full ridge regression approach. As recalled by^32^, whereas the LASSO approach allows to shrink and select, the EN procedure not only shrinks and selects but also makes sure that the selected model also minimizes the Kullback-Liebler distance to the true DGP (thus meeting the so-called ‘oracle property’). In our implementation, as done in the applied literature in various fields (see^32^ or^33^ to quote only a few) we consider a value of 0.5 for $\alpha$, which allows us to perform an equally weighted approach between ridge regression and LASSO. Finally, the $\lambda$ parameter controls the strength of the penalty, that is, the number of covariates set to zero (if $\alpha \ne 0$) or shrink toward zero (if $\alpha =0$) for minimization purposes.

The regression framework is convenient to assess the role of local environment information as opposed to traditional global environment information for computing topological metrics and analyzing systemic risk. For the sake of clarity, we can more specifically formulate three testable hypotheses.

- **H1**: Information regarding systemic exposure of firms is concentrated at the **local level**. If so, information coming from the remaining part of the network only adds noise and should be filtered out.
- **H2**: Information regarding systemic exposure of firms in the local environment is entirely embodied in the **global environment**.
- **H3**: Both **local and global levels** embed non-overlapping information.

If **H1** is true, only LT metrics should appear significant from a regression including both local and global topological metrics. If **H2** is true, only GT should emerge as significant. If **H3** is true, both LT and GT variables should appear significant when included in the same model.

## Results

Before interpreting the regression results, we briefly discuss the cross-correlations among our selected topological metrics. Exploring the dependence among all our metrics may help to have a better sense of their informational content; for example, a very strong correlation would suggest the absence of specific information in an isolated variable. Next, we estimate a baseline model, which is limited to traditional centrality metrics computed at the network level. Then, we extend the specification to include community-based metrics. Eventually, we include all the variables in the model. This stepwise procedure aims to assess the robustness of our findings.

### The correlation matrix

**Figure 4:**
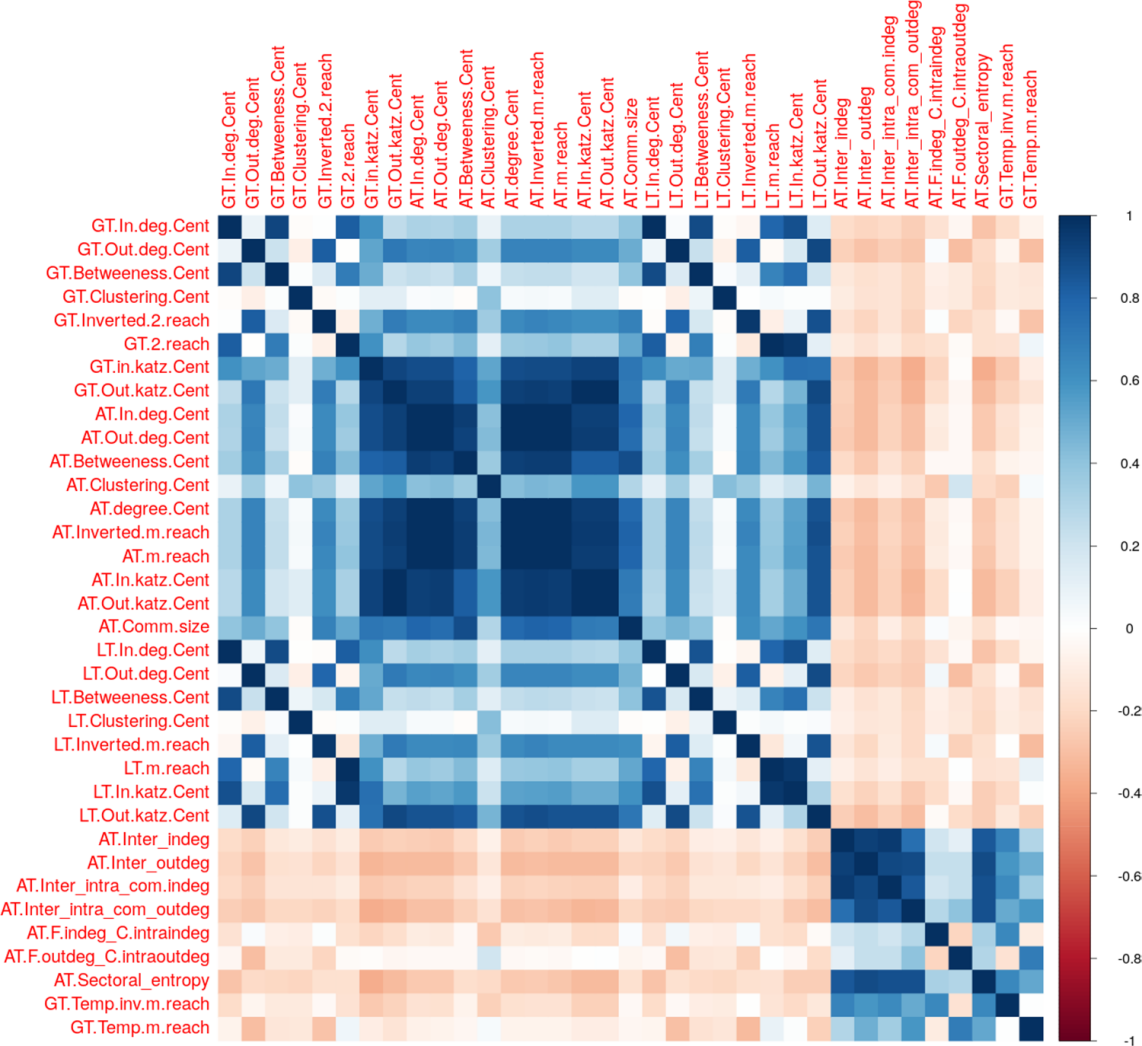
Correlation matrix of topological metrics. The table displays the correlation matrix among the centrality measures.

We use a heatmap to display the correlation coefficients (see Fig. 4). Dark colors signal strong correlations. Positive correlations are reported in blue and negative ones in red. We can visually separate two groups of variables. The first group embeds most of the traditional topological metrics computed either at the network or the community level. Overall, the variables display positive correlations with nuances regarding their strength as the correlation coefficients range from 0.1 to 0.9. The positive signs suggest that the various measures embed consistent information regarding the centrality of institutions within the network. While consistent, this information does not fully overlap. A second group includes less conventional measures such as sectoral entropy or temporal m-reach centrality measures. The correlation values are now weak and slightly negative in terms of the majority of other variables. The results also support the interest of considering a wide variety of centrality indicators as they do not share the same information. Whether they are informative to predict financial vulnerability remains an open question that can be addressed via regression analyses.

### First level: Global topological metrics

We first report the results for the regular metrics. In total, the model includes 8 variables, all computed at the system-wide level. The list of variables can be found in Fig. 2. We just omit the two temporal (GT) variables in this baseline model. The rows in Table 1 report the names along with the signs of the coefficient of the variables that appear significant in one of the four columns. Each column corresponds to an alternative definition of the dependent variable. We consider four alternatives depending on the definition of both the crisis period, a period of either 12 or 18 months, and the vulnerability of the firm, computed either as cumulative returns or maximum drawdown. Four variables are identified as significant regardless of how the dependent variable has been computed with the exception of the *out-degree* variable, which is not significant in one model. As expected, the signs for the cumulative returns and the maximum drawdown are opposite. Higher vulnerability corresponds to smaller cumulative returns and larger maximum drawdown. To illustrate our results, we can take the example of the *betweenness centrality* metric. Note that *betweenness centrality* is the number of shortest paths that pass through a node. In this context, each edge represents the existence of spillovers between the asset returns of two financial institutions. The path means that distress in one institution can be channeled to another in the network through indirect connections. The *betweenness centrality* metric therefore captures whether a financial firm is located in the path of these financial spillovers. It can also be viewed as a measure of the capability of a firm to transmit the crisis between separated groups of firms. Our results support that the higher the number of paths passing through a firm, the greater the losses experienced by this firm during the crisis and thus, the more vulnerable it appears to be.

**Table 1:** Penalized multivariate regressions for system wide firm-based metrics (GT).

Selected variables	C.R:12 m	C.R:18 m	M.D:12 m	M.D:18 m
Out-degree centrality (GT)	$+$	$+$		−
Betweenness centrality (GT)	−	−	$+$	$+$
Clustering (GT)	−	−	$+$	$+$
In-Katz centrality (GT)	−	−	$+$	$+$

−, + denote the sign of the coefficient for significant variable. The variables not significant are not reported. The model is estimated by using Elastic Net regression. Each column corresponds to alternative definition of the dependent variable. In columns 1 and 2, the dependent variable is computed as cumulative returns over respectively 12 months and 18 months. In columns 3 and 4, the dependent variable is computed as the maximum drawdown over 12 months and 18 months, respectively.

### Second level: global and aggregated topological metrics

In the second estimation, we add all the variables averaged by community (AT) to the baseline model (GT). By doing so, we can assess the stability of the firm-based results along with the community-level analysis. Results are depicted in Table 2. The traditional metrics appear robust when compared to the first-level estimations. Only three changes are noticeable. The *out-degree* variable is now significant with respect to the four alternative definitions of the dependent variable, which previously appeared as significant in three models. The *betweenness centrality* is no more significant when we consider a long period of 18 months for the crisis and the maximum drawdown to quantify firm losses. Eventually, *in-Katz centrality* is no more significant when using an 18-month crisis period and the cumulative returns. Among the 10 variables computed at the community level, three are significant once traditional system-wide metrics, *clustering*, *2-reach* and *community size*, are controlled for. This result means that the larger a community, the more vulnerable a firm is, for example.

**Table 2:** Penalized multivariate regressions for system wide firm-based (GT) metrics and community-based metrics (AT).

Selected variables	C.R:12 m	C.R:18 m	M.D:12 m	M.D:18 m
Out-degree centrality (GT)	$+$	$+$	−	−
Betweenness centrality (GT)	−	−	$+$	
Clustering centrality (GT)	−	−	$+$	$+$
In-Katz centrality (GT)	−		$+$	$+$
Clustering (AT)	−	−	$+$	
2-reach centrality (AT)	−	−		
Community size (AT)	−		$+$	$+$

−, + denote the sign of the coefficient for significant variable. The variables not significant are not reported. The model is estimated by using Elastic Net regression. Each column corresponds to alternative definition of the dependent variable. In columns 1 and 2, the dependent variable is computed as cumulative returns over respectively 12 months and 18 months. In columns 3 and 4, the dependent variable is computed as the maximum drawdown over 12 months and 18 months, respectively.

### Third level: complete model

We can now consider the full model. The specification includes the three categories of variables, adding community firm-based metrics (noted as LT in Fig. 2) to the previous model (noted as GT and AT in Fig. 2). We also add our set of alternative metrics (noted as new-GT and new-AT in Fig. 2). As explained in Step 2 of the methodology, these variables exploit the information embedded in the temporal changes in the network, the sectoral diversity of our financial firms, as well as the intra- and extra-community links. The results are reported in Table 3. System-wide metrics are stable for *betweenness* and *clustering*. Out-Katz centrality performs better than the out-degree variable adding up the influence of the neighboring nodes. On the other hand, inverted 2-reach centrality replaced the in-Katz centrality to capture financial vulnerability. The size of the community remains significant, as expected and explained in the previous section. Turning to the community firm-based (LT) metrics, two variables expressing outgoing and incoming links are the dominant explanatory variables at this level. The first is the out-degree centrality. Interestingly, we have previously noticed that this variable was significant when computed at the network level. Now that the two variables computed at the network and the community level are competing in the same model, the one at the community level emerges as significant. The second variable is the inverted 2-reach centrality. We note that in this case, the same metrics computed at both the system-wide and the community level are significant while included in the same model. Among the set of alternative metrics, five variables appear as significant: inter-intra-outdegree centrality, outdeg-intra-outdeg, sectoral entropy, temporal inverted 2-reach, and temporal 2-reach. Recall that *inter-intra-out-degree centrality*, is defined as the ratio between the community out-degree to the other communities in the network and the out-degree within the same community. What we want to express by such a metric is the influence that one community has over the whole system, normalized by the own intra-dependence. The higher the variable, the more vulnerable the firm. The second metrics is the outdeg-intra-outdeg. The creation of this metric was inspired by^26^, defined as the node’s commitment with its own community. Here, the metric is defined as the ratio between the node’s out-degree and the total out-degree within its community. The sign associated with the first column when the dependent variable is constructed as cumulative returns is positive. The sign is negative when considering the maximum drawdown. This result means that the higher the variable, the less vulnerable the firm. The *sectoral entropy* captures the diversity of sectors within the community. In the descriptive analysis of^20^, sectoral diversity was suggested to be a determinant systemic risk when computed as the global sector-interface. Our estimations based on a formal regression analysis confirm this feature. However, it is worth noting that the metric is sensitive to the time length, appearing as significant only for the shortest crisis period (12 months). Finally, we have the temporal versions of the m-reach centrality. Only the one considering outgoing links (temporal 2-reach) is dominant. This last result highlights the importance of considering higher-order metrics in systemic risk^34^.

**Table 3:** Penalized multivariate regressions for system wide firm-based metrics, community-based metrics and community firm-based metrics.

Selected variables	C.R:12 m	C.R:18 m	M.D:12 m	M.D:18 m
Betweenness centrality (GT)	−	−	$+$	$+$
Clustering (GT)	−	−	$+$	$+$
Inverted 2-reach centrality (GT)	$+$	$+$	−	−
Out-Katz centrality (GT)	−	−	$+$	$+$
Community size (AT)			$+$	$+$
Out-degree centrality (LT)	$+$	$+$	−	−
Inverted 2-reach centrality (LT)	$+$	$+$	−	
Inter-intra-outdeg (new-AT)	−		$+$	
Outdeg-intra-outdeg (new GT)	$+$	$+$		−
Sectoral-entropy (new-AT)	$+$		−	
Temporal inverted 2-reach (new GT)				$+$
Temporal 2-reach (new GT)	−	−	$+$	$+$

−, + denote the sign of the coefficient for significant variable. The variables not significant are not reported. The model is estimated by using Elastic Net regression. Each column corresponds to alternative definition of the dependent variable. In columns 1 and 2, the dependent variable is computed as cumulative returns over respectively 12 months and 18 months. In columns 3 and 4, the dependent variable is computed as the maximum drawdown over 12 months and 18 months, respectively.

### Non-penalized regression analysis

As a last exercise, we re-estimate the model using ordinary least squares (OLS). Penalization methods such as LASSO or EN offer relevant solutions to select acute sets of predictors for small sample sizes relative to the number of variables. While regular OLS regression minimizes the sum of squared residuals to find the value of the estimated coefficients, penalized regressions augment the minimization problem using a penalty term. As a result, penalized regression leads to an increase in the estimation bias by shrinking coefficients, with non-relevant ones becoming (nearly) zero. To check the robustness of our results, we apply OLS on the full set of the selected regressors. Under regular conditions (i.e., Gauss-Markov hypotheses) OLS estimators are unbiased. This two-step procedure can be of particular interest to specifically check the sign of the coefficients. However, statistical tests can still suffer from low power due both to the limited degree of freedom despite having dropped some regressors and to multicollinearity among the remaining ones. Neither the shrinkage approach nor the linear regression is a panacea. However, cross validating their results can allow us to be more confident in our conclusions. The estimated coefficients along with robust standard errors are reported in Table 4. Overall, our findings are confirmed. We do not observe reversals in signs. For most variables in each category, they remains significant predictors of a firm’s vulnerability when considering the 10% significance level. In a few cases, the variables are significant in a lower number of models. In three cases, inverted 2-reach centrality, sectoral entropy, and temporal inverted 2-reach, we can no longer detect statistical significance at a 10% level in the four specifications.

**Table 4:** Non-penalized multivariate regressions for system wide firm-based metrics, community-based metrics and community firm-based metrics.

Name of regressors	C.R:12 m	C.R:18 m	M.D:12 m	M.D:18 m
Cst	− 0.17	− 0.30***	0.48***	0.51***
	(0.12)	(0.07)	(0.08)	(0.06)
Betweenness centrality (GT)	− 5.54***	− 4.72***	3.19***	4.01***
	(1.12)	(0.99)	(0.87)	(0.73)
Clustering (GT)	− 0.41***	− 0.44***	0.32**	0.13
	(0.15)	(0.12)	(0.15)	(0.09)
Inverted 2-reach centrality (GT)	− 0.00	− 0.00	0.00	− 0.00***
	(0.00)	(0.00)	(0.00)	(0.00)
Out-Katz centrality (GT)	− 0.19***	− 0.16***	0.07*	0.12***
	(0.05)	(0.04)	(0.03)	(0.03)
Community size (AT)	–	–	0.00**	0.00
			(0.00)	(0.00)
Out-degree centrality (LT)	0.00***	0.00***	− 0.00**	− 0.00***
	(0.00)	(0.00)	(0.00)	(0.00)
Inverted 2-reach centrality (LT)	0.01	0.00	− 0.00	–
	(0.00)	(0.00)	(0.00)	
Inter-intra-outdeg (new-AT)	− 0.03*	–	− 0.00	–
	(0.01)		(0.00)	
Outdeg-intra-outdeg (new GT)	0.02***	0.01*	–	− 0.01**
	(0.00)	(0.00)		(0.00)
Sectoral-entropy (new-AT)	0.02	–	− 0.01**	–
	(0.01)		(0.01)	
Temporal inverted 2-reach (new GT)	–	–	–	0.00
				(0.00)
Temporal 2-reach (new GT)	− 0.00***	− 0.00***	0.00***	0.00***
	0.00	(0.00)	(0.00)	(0.00)
Adjusted $R^2$	41	41	27	38
N. obs.	46	46	46	46

This table reports the cross-sectional regression of alternative measures of financial firms vulnerability on centrality measures selected in Table 3. The dependent variable is (i) the cumulative returns over 12 months in column 2, (ii) the cumulative returns over 18 months in column 3, (iii) the maximum drawdown over 12 months in column 4, (iv) the maximum drawdown over 18 months in column 5. Estimation is performed by ordinary least squares. Robust standard error are reported in parentheses. ***, **, * stand for statistical significance at 1%, 5% and 10% levels.

## Supplementary information

Supplementary Information.

## References

[1] Temizsoy A, Iori G, Montes-Rojas G (2017). Network centrality and funding rates in the e-mid interbank market. J. Financial Stab..

[2] Geraci M, Gnabo J-Y (2018). Measuring interconnectedness between financial institutions with Bayesian time-varying vector autoregressions. J. Financial Quant. Anal..

[3] Glasserman P, Young HP (2016). Contagion in financial networks. J. Econ. Lit..

[4] Boss M, Elsinger H, Summer M, Thurner S (2004). Network topology of the interbank market. Quant. Finance.

[5] Santos, E. & Cont, R. The Brazilian interbank network structure and systemic risk. Working Papers Series 219, Central Bank of Brazil, Research Department (2010).

[6] Cont R, Moussa A, Santos EB, Langsam JFJ (2013). Network structure and systemic risk in banking systems. Handbook of Systemic Risk.

[7] Caldarelli G, Battiston S, Garlaschelli D, Catanzaro M (2004). Emergence of Complexity in Financial Networks.

[8] Craig B, von Peter G (2014). Interbank tiering and money center banks. J. Financial Intermed..

[9] Newman M (2010). Networks: An Introduction.

[10] Craig, B., Koetter, M. & Krüger, U. *Interbank lending and distress: Observables, unobservables, and network structure*. Tech. Rep. 2014.

[11] Martinez-Jaramillo S, Alexandrova-Kabadjova B, Bravo-Benitez B, Solórzano-Margain JP (2014). An empirical study of the Mexican banking system’s network and its implications for systemic risk. J. Econ. Dyn. Control.

[12] Puhr, C., Seliger, R. & Sigmund, M. Contagiousness and Vulnerability in the Austrian Interbank Market. *Financial Stability Report* 62–78, (2012).

[13] Poledna S, Molina-Borboa JL, Martinez-Jaramillo S, van der Leij M, Thurner S (2015). The multi-layer network nature of systemic risk and its implications for the costs of financial crises. J. Financial Stab..

[14] Gould DM, Kenett DY, Panterov G (2020). Multi‐dimensional economic connectivity: benefits, risks, and policy implications. Int. J. Fin. Econ..

[15] Cai F, Zheng L (2004). Institutional trading and stock returns. Finance Res. Lett..

[16] Billio M, Getmansky M, Lo AW, Pelizzon L (2012). Econometric measures of connectedness and systemic risk in the finance and insurance sectors. J. Financial Econ..

[17] Diebold FX, Yilmaz K (2014). On the network topology of variance decompositions: Measuring the connectedness of financial firms. J. Econom..

[18] Giudici P, Parisi L (2019). Bail-in or bail-out? Correlation networks to measure the systemic implications of bank resolution. Risks.

[19] Betz F, Hautsch N, Peltonen TA, Schienle M (2016). Systemic risk spillovers in the European banking and sovereign network. J. Financial Stab..

[20] Gandica Y, Geraci MV, Béreau S, Gnabo J-Y (2018). Fragmentation, integration and macroprudential surveillance of the us financial industry: Insights from network science. PLOS ONE.

[21] Adrian, T. Measuring risk in the hedge fund sector. *Curr. Issues Econ. Finance* **13**, (2007).

[22] Korobilis, D. & Yilmaz, K. Measuring dynamic connectedness with large bayesian VAR models. Essex Finance Centre Working Papers, University of Essex (2018).

[23] Wang, D., van Lelyveld, I. & Schaumburg, J. Do information contagion and business model similarities explain bank credit risk commonalities? Tinbergen Institute Discussion Papers 18-100/IV, Tinbergen Institute (2018).

[24] Balla E, Ergen I, Migueis M (2014). Tail dependence and indicators of systemic risk for large US depositories. J. Financial Stab..

[25] Blondel V, Guillaume J-L, Lambiotte R, Lefebvre E (2008). Fast unfolding of communities in large networks. J. Stat. Mech..

[26] Palla G, Barabási A-L, Vicsek T (2007). Quantifying social group evolution. Nature.

[27] Zou H, Hastie T (2005). Regularization and variable selection via the elastic net. J. R. Stat. Soc. Ser. B Stat. Methodol..

[28] https://www.rdocumentation.org/packages/glmnet/versions/2.0-16/topics/glmnet.

[29] https://web.stanford.edu/~hastie/papers/glmnet~vignette.pdf.

[30] Tibshirani R (1996). Regression shrinkage and selection via the lasso. J. R. Stat. Soc. Ser. B Methodol..

[31] Hoerl AE, Kennard RW (1970). Ridge regression: Biased estimation for nonorthogonal problems. Technometrics.

[32] Demirer M, Diebold FX, Liu L, Yilmaz K (2018). Estimating global bank network connectedness. J. Appl. Econ..

[33] Gonzales G, De Saeger S (2018). Elastic net regularized regression for time-series analysis of plasma metabolome stability under sub-optimal freezing condition. Sci Rep.

[34] Lambiotte R, Rosvall M, Scholtes I (2019). From networks to optimal higher-order models of complex systems. Nat. Phys..

