# Supplementary material for: A multilevel analysis of financial institutions’ systemic exposure from local and system-wide information
Source: Sci Rep. 2020 Oct 19;10:17657. doi: 10.1038/s41598-020-74259-7 (PMC7573582; doi:10.1038/s41598-020-74259-7)
Supplement: Supplementary file 1 — Supplementary Information. [file 41598_2020_74259_MOESM1_ESM.pdf]

Supplementay Information:
A multilevel analysis of financial institutions' systemic exposure from local and system-wide information

Yérali Gandica [1,2,3,4,5], Sophie Béreau [1,2,3], Jean-Yves Gnabo [1,2]

1. Center for Research in Finance and Management (CeReFiM), University of Namur, Belgium
2. Namur Institute for Complex Systems (naXys), University of Namur, Belgium
3. Center for Operations Research and Econometrics (CORE), Université catholique de Louvain, Belgium
4. Institute of Information and Communication Technologies, Electronics and Applied Mathematics (ICTEAM), Université catholique de Louvain, Belgium
5. CY Cergy Paris Université, CNRS, Laboratoire de physique théorique et modelisation. F95000 Cergy. Île-de-France. France.
Corresponding author. E-mail: ygandica@gmail.com

SA.1. Methodology

The network used in this analysis is similar to[1] and relies on the approach developed by[2]. In what follows, we review the building blocks of the methodology of[2].

First, the approach builds on the notion of Granger causality. Granger causality attempts to infer causality using statistics. A primary requirement for the notion to be applied is that the variables of interest must be measurable and observable times series. Let $x = \{x_t\}_{t=0}^{T}$ and $y = \{y_t\}_{t=0}^{T}$ be two stationary time series, where $t = 0$ stands for the origin date of the data, and $t = T$ its ultimate observation. In our context, those series are assumed to be the log-returns of the price indices related to the different firms or our sample. By definition, x causes y in the sense of[3] if the past of x can improve the forecast of y based on the past of y alone. The definition rests on the central principle according to which *the cause must occur prior to the effect*. Second, an econometric strategy is needed to test the existence of Granger causality between two series on data.[4] proposes to transpose the definition to a simple econometric set up to test the Granger causality up to a lag truncation p, by the following two regressions estimated by ordinary least squares (OLS):

$$\begin{aligned} y_t &= \hat{\alpha} + \sum_{s=1}^{p} \hat{\phi}_s y_{t-s} + \sum_{s=1}^{p} \hat{\beta}_s x_{t-s} + \hat{\varepsilon}_t^U \\ y_t &= \hat{\alpha} + \sum_{s=1}^{p} \hat{\beta}_s x_{t-s} + \hat{\varepsilon}_t^R \end{aligned} \tag{1}$$

with $SSR^R = \sum_{s=1}^{T} (\hat{\varepsilon}_t^R)^2/T$ and $SSR^U = \sum_{s=1}^{T} (\hat{\varepsilon}_t^U)^2/T$. An associated measure of linear feedback from

X to Y (or of the strength of Granger causality from x to y) is given by: Granger-Wald statistic as follows:

$$\hat{F}_{x \to y} = ln(SSR^U / SSR^R) \tag{2}$$

If the underlying errors in equations 1 and 2 are independent and identically distributed, $T\hat{F}_{x \to y}$ is known to have an asymptotic χ^2 distribution with p degrees of freedom under the null hypothesis that $T\hat{F}_{x \to y} = 0$. The latter is equivalent to testing the joint hypotheses on the parameters of the unrestricted model in equation 1:

$$H_0 : \beta_1 = \beta_2 = \cdots = \beta_p = 0 \tag{3}$$

In the network literature, Granger causality has been often used to infer causal networks (see[5] for an example). Specifically, a directed edge between two nodes (representing two time series) is drawn if the null hypothesis of classical Granger non-causality shown in equation 4 is rejected. Hence, for every pair of nodes $x, y \in V$, there is a link from node x to y if the set of coefficients associated with the past of x in a regression of y on its own past and on the past of x, are jointly significantly different from zero. In the case of no Granger causality, there is no edge between x and y while if y Granger causes x and x Granger causes y then there is a bi-directional edge between x and y.

Third, the notion of Granger causality can be generalised to account for changes in the direction and intensity of causality. To that aim,[2] propose to adopt a time-varying parameters approach and test whether the causal relation holds in every moment in time. The time-varying parameters representation that allows for the relationship between y and x to vary through time can be written as follows:

$$y_t = \alpha_t + \sum_{s=1}^{p} \phi_{s,t} y_{t-s} + \sum_{s=1}^{p} \beta_{s,t} x_{t-s} + \hat{\varepsilon}_t^U \tag{4}$$

Fourth, the estimation of the time-varying version of the model is done by implementing the Kalman filter and smoother. Equation 5 can be considered as the measurement equation of a state space model. In

particular, by stacking the parameters into a vector θ_t, equation 5 can be rewritten in a compact-form as:

$$y_t = X_t^{'} \theta_t + u_t, \tag{5}$$

Where X_t contains a constant and the regressors i.e., the lags of y_t and x_t. Errors u_t are assumed to be Normally distributed, with mean zero and constant variance-covariance matrix Σ. The original model of[2] allows for heteroskedasticity and fat-tailed errors. In our network, however, a simpler approach was adopted consisting in standardizing the log-returns in a first step, that is computing $y_t = \frac{r_t - \bar{r}}{\widehat{\sigma}_{r,t}}$, with r_t the log-returns calculated on our price indices, $\bar{r}$ the sample mean, and $\widehat{\sigma}_{r,t}$ the estimated time-varying standard deviation (volatility) component according to a GARCH(1,1) specification. Similarly to several macroeconomic studies (e.g.[6,7]), it is assumed that parameters evolve according to a driftless random walk. The state equation of the model is thus given by:

$$\theta_t = \theta_{t-1} + \nu_t, \tag{6}$$

where ν_t is Normal with mean zero and variance Q.

Fifth, testing was then based on using the simulated distributions to estimate the probability of the states satisfying the null hypothesis specified in equation 6. More precisely we calculate the Savage-Dickey density ratio which is a convenient form of the Bayes factor. The Bayes factor, gives the odds in favor of the null hypothesis against the alternative hypothesis, $H_{1,t} : B_t^{(ji)} \neq 0$, without assuming that the null hypothesis is true. It is estimated following[8]. Once retrieved, the Bayes factor gives a direct mapping to the implied probability that the null is true. Note that if $\hat{K}_t^{ji}$ is the Bayes factor for $H_{0,t}^{(ji)}$, then the implied probability that the null is true is just $\hat{K}_t^{ji}/(1+\hat{K}_t^{ji})$. Edges between nodes are thus confirmed if the implied probability that the null is true can be rejected.

References

1. Gandica, Y., Lambiotte, R. & Carletti, T. What can wikipedia tell us about the global or local character of burstiness? *The Work. Tenth Int. AAAI Conf. on Web Soc. Media Wiki: Tech. Rep. WS-16-17* (2016).

2. Geraci, M. & Gnabo, J.-Y. Measuring interconnectedness between financial institutions with bayesian time-varying vector autoregressions. *J. financial quantitative analysis* **53**, 1371–1390 (2018).

3. Granger, C. Investigating causal relations by econometric models and cross-spectral methods. *Econometrica* **37**, 424–438 (1969).

4. Geweke, J. Measurement of linear dependence and feedback between multiple time series. *J. Am. Stat. Assoc.* **77**, 304–313 (1982).

5. Billio, M., Getmansky, M., Lo, A. W. & Pelizzon, L. Econometric measures of connectedness and systemic risk in the finance and insurance sectors. *J. Financial Econ.* **104**, 535–559 (2012).

6. Cogley, T. & Sargent, T. Drifts and volatilities: Monetary policies and outcomes in the post wwii us. *Rev. Econ. Dyn.* **8**, 262–302 (2005).

7. Primiceri, G. Time varying structural vector autoregressions and monetary policy. *Rev. Econ. Studies,* **72**, 821–852 (2005).

8. Koop, G., Leon-Gonzalez, R. & Strachan, R. W. Dynamic probabilities of restrictions in state space models: An application to the phillips curve. *J. Bus. Econ. Stat.* **28**, 370–379 (2010).
